# The cerebral mechanism of acupuncture for treating knee osteoarthritis: study protocol for a randomized controlled trial

**DOI:** 10.1186/s13063-019-3233-7

**Published:** 2019-02-13

**Authors:** Jing Guo, Yang Chen, Zhengjie Li, Shirui Cheng, Chenjian Tang, Xiaohui Dong, Wenhua He, Yong Huang, Bao Yin, Yu Sheng, Jun Zhou, Aijia Li, Fang Zeng, Lei Lan, Fanrong Liang

**Affiliations:** 1 Acupuncture and Tuina School/The 3rd Teaching Hospital, Chengdu University of Traditional Chinese Medicine, 37# Shierqiao Road, Chengdu, 610075 Sichuan China; 2 The 1st Teaching Hospital of Chengdu University of Traditional Chinese Medicine, Chengdu, Sichuan China

**Keywords:** Acupuncture, Knee pain, Central mechanism, Functional magnetic resonance imaging

## Abstract

**Background:**

Acupuncture is safe and effective for reducing the symptoms of knee osteoarthritis (KOA), but the underlying mechanisms of acupuncture for treating KOA are not fully understood.

**Methods/design:**

In total, 108 participants diagnosed with KOA will be recruited. They will be blinded to group assignment and randomized to either verum acupuncture, sham acupuncture or waiting-list groups with 36 patients in each group. Each patient in the acupuncture group will receive five treatments per week for 2 weeks. This study will focus on detecting the cerebral functional connectivity changes elicited by acupuncture treatment. The Visual Analog Scale and the short form of the McGill Pain Questionnaire, the Western Ontario and McMaster Universities Osteoarthritis Index, the Attention Test Scale, the Pain Assessment of Sphygmomanometer and the 12-Item Short Form Health Survey will be used to evaluate the symptoms and quality of life improvement at the baseline and the end of treatment. The Self-rating Anxiety Scale and the Self-rating Depression Scale will be used at the baseline and the end of treatment to investigate the influence of emotional state on brain activity and clinical variable. To ensure the consistency of acupuncture manipulation, the *deqi* scale will be performed after each acupuncture treatment. During the procedure of outcome evaluation and data analysis, the evaluators and statisticians will be blinded to the group allocation. The repeated measures analysis of variance (3 groups × 2 time points ANOVA) will be employed to analyze numerical variables of the clinical and neuroimaging data generated in the study, then the *t* test will be used in the post-hoc analysis.

**Discussion:**

The results of this randomized, sham- and waiting-list-controlled functional magnetic resonance imaging (fMRI) study will help to investigate the influence of verum acupuncture treatment on the brain activities of patients with KOA, which might provide evidence for the clinical application of verum acupuncture for KOA management.

**Trial registration:**

Chinese Clinical Trial Registry, ID: ChiCT-IOR-17012364. Registered on 14 August 2017.

## Background

Knee osteoarthritis (KOA) is characterized by the degeneration of cartilage in the knee, manifesting as knee pain, function limitation and so forth [1]. As a high-prevalence chronic joint disease [2], KOA has become one of the leading causes of disability in the world and brings an increasing social and economic burden [3, 4]. Furthermore, the pharmacological treatment of KOA, such as the first-line treatment NSAIDs (non-steroidal anti-inflammatory drugs), is unsatisfactory with significant side effects [5].

In China and some other Asian countries, the history of using acupuncture for treating knee pain caused by osteoarthritis can be dated back thousands of years. In Western countries, acupuncture has been gradually accepted as a promising treatment option for KOA. Recently, both clinical studies and systematic reviews have shown that acupuncture is safe and effective for KOA in pain relief [6–8], stiffness [9] and physical function improvement [10] with a low risk of adverse reactions. Although one study has reported that acupuncture can alleviate weight-bearing problems [11], it is considered more economical for improving quality-adjusted life years in the treatment of KOA, compared with NSAIDs [12]. However, the underlying mechanisms of acupuncture for treating KOA remains unclear, which prevents its wider application in clinical practice.

KOA is a complex, chronic pain condition partly due to both nociceptive and neuropathic mechanisms [13]. Previous studies have demonstrated that central sensitization, a pathological change in the central nervous system, plays an important role in the pathogenesis of KOA [14, 15]. With the widely application of neuroimaging techniques in pain research, investigators not only mapped the brain regions which are involved in the central pathogenesis of chronic pain, but also partly explored the central mechanism of acupuncture-induced analgesia by detecting the cerebral responses elicited by acupuncture. Using resting-state functional magnetic resonance imaging (rs-fMRI), researchers found that the patients with chronic pain demonstrated altered functional connectivity (FC) of the medial prefrontal default-mode network (DMN) and a changed regional homogeneity (ReHo) value of the anterior cingulate cortex (ACC) [16–18]. These results greatly enhanced the understanding of the central mechanism of chronic pain and have provided an approach to investigating the mechanism of acupuncture-induced analgesia. For instance, a functional magnetic resonance imaging (fMRI) study has shown that acupuncture can regulate the descending pain-modulatory system of KOA patients by enhancing the FC between the right frontoparietal network (rFPN) and the medial prefrontal cortex (mPFC) [19]. However, the sample size (only 10 in each group) and the dosage of acupuncture treatment (six acupuncture treatments for 4 weeks) of that study were relatively small, and the self-healing tendency of KOA patients could not be excluded due to the lack of waiting-list control.

So, this study aims to (1) investigate the influence of verum acupuncture treatment on the brain activities of patients with KOA compared with that of sham acupuncture treatment and waiting list patients by using fMRI with a larger sample size (36 patients in each group) and (2) analyze the possible correlations between the changes of cerebral activity and the improvement of clinical variables in each group, so as to explore how acupuncture manages pain by modulating brain function.

## Methods/design

### Study design

**Fig. 1:**
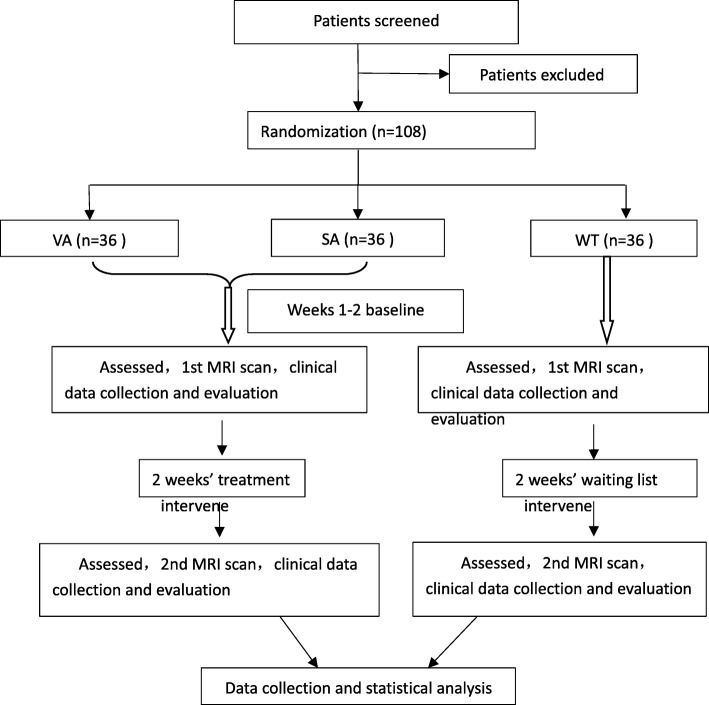
Flow chart of the trial. The present study is a randomized controlled neuroimaging trial. One hundred and eight knee osteoarthritis (KOA) patients will be included and randomized equally to one of three groups: a verum acupuncture group, a sham acupuncture group and a waiting-list group. For the 36 patients in each group, this trial will include a 2-week baseline period and a 2-week treatment period. During the 2-week treatment, patients in the two acupuncture groups will receive 10 sessions of puncturing treatments, while the waiting-list group will not receive acupuncture. Both the outcome assessments and functional magnetic resonance imaging (fMRI) scan will be performed at two time points: baseline and end of acupuncture treatments. The central mechanism of verum acupuncture in the treatment of KOA will be analyzed after data collection

**Fig. 2:**
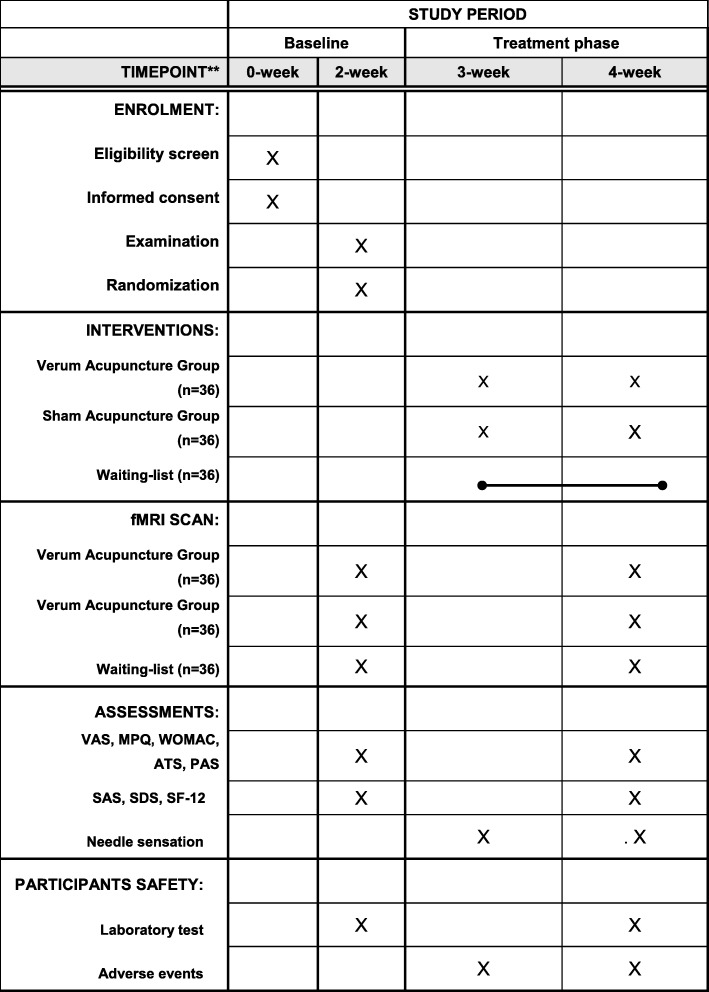
Study schedule for data collection. The informed consent and examination will be conducted after recruitment. Then, matched KOA patients will be randomized into three groups, only two acupuncture groups will receive treatment. Both clinical outcomes and functional magnetic resonance imaging (fMRI) scans will be performed at two time points including: the baseline and the end of acupuncture treatments. Adverse events will be recorded in the case report form at any time during the study. *VAS* Visual Analogue Score, *MPQ* McGill Pain Questionnaire, *WOMAC* Western Ontario and McMaster University Osteoarthritis Index, *ATS* Attention Test Scale, *PAS* Pain Assessment of Sphygmomanometer, *SAS* Self-Rating Anxiety Scale, *SDS* Self-Rating Depression Scale, *SF-12* Short Form 12 Health Survey

This is a randomized controlled, parallel neuroimaging study, with participants and assessor blinded to the group assignment. A total of 108 participants diagnosed with KOA in accordance with American College of Rheumatology (ACR) criteria (1991 revised version) [20] will be considered as eligible patients. They will be randomly allocated into three equal groups with 36 patients in each group, including a verum acupuncture group, a sham acupuncture group and a waiting-list group. The treatment period will last for 2 weeks. Outcome measurements and MRI scan will be assessed at baseline and at the end of treatment (Figs. 1 and 2). The changes of clinical variables and cerebral activity of each group will be analyzed after data collection.

#### Sample size

In general, 12 to 15 patients per group provided statistical power in MRI studies [21]. According to our previous studies, 25 patients in each group has a more stable statistical power for brain functional network analysis [22]. In order to have a powerful and repeatable statistical power, we require 30 patients per group in this trial. Considering a 20% dropout rate and the possible excessive head motion during scanning, we will include 36 KOA participants in each group. Finally, we plan to enroll 108 participants and each group will undergo two MRI scans to investigate the different central responses among verum acupuncture, sham acupuncture and waiting-list treatment in KOA patients with pain.

#### Randomization

Eligible patients will be randomized in a ratio of 1:1:1 to the verum acupuncture group, sham acupuncture group and waiting-list group with 36 patients in each group using simple randomization. Random number lists will be created in accordance with PROC PLAN of SAS 9.2 (SAS Institute Inc., Cary, NC, USA). The randomization allocation will use sequentially numbered, opaque and sealed envelopes by an independent assistant.

#### Blinding

Acupuncturists, who will be asked to apply the same method of stimulation, will not be blinded to treatment allocation for the different location in each group. And the acupuncture treatment will be revealed to the acupuncturists before treatment starts. However, participants will be blinded to group assignment. They will be informed that there are two types of acupuncture treatment provided in this study. They will accept one of them in a separate compartment. Each of the patients only knows the type which they accepted but not the other type. As the waiting-list group is without any intervention, it is impossible to blind the patients, clinicians and investigators. The evaluators and statisticians will be blinded to the group allocation in the outcome evaluation procedure and data analysis for the sake of reducing the risk of bias.

### Participants

Knee-pain patients who suffered from KOA according to the American College of Rheumatology (ACR) criteria (1991 revised version) [20] will be recruited from outpatient and inpatient departments of the First Affiliated Hospital and the Third Affiliated Hospital of Chengdu University of Traditional Chinese Medicine. Potential patients will also be recruited through posters, the Internet and leaflets.

#### Inclusion criteria

Inclusion criteria require that all patients: (1) meet the diagnostic criteria for KOA set by the ACR in 1991 [20], including retropatellar, medial or lateral KOA, etc., with no traumatic aetiology, (2) are aged between 40 and 60 years and right-handed, (3) have not taken any pain-killing medicine within 1 month, (4) have not received any acupuncture treatment within 1 month, (5) have had at least 3 months of knee-pain duration and (6) have an average knee-pain score on a Visual Analog Scale (VAS) ≥ 3 (range from 0 to 10) in the past 2 weeks, (7) have I–II degree knee-joint radiological change on the Kellgren-Lawrence scale and (8) have signed an informed consent.

#### Exclusion criteria

Patients will be excluded if they: (1) are alcohol or drug abusers or are taking other medications that may influence brain-imaging outcomes or (2) are pregnant or lactating women or (3) are suffering from psychiatric, neurological, gastrointestinal, cardiovascular, infectious, immunological, respiratory or renal illnesses or (4) are suffering from any other chronic pain condition or have a history of head trauma with loss of consciousness or (5) are diagnosed as rheumatoid arthritis or other leg-related pain disorders or (6) have MRI contraindications such as claustrophobia, cardiac pacemaker, defibrillator, heart stenting, intrauterine device or (7) have acupuncture contraindications such as a bleeding tendency.

#### Informed consent

The authors retain full control of the manuscript content. All patients will be informed of the random allocation of verum acupuncture, sham acupuncture or waiting-list treatment and the possible benefits and risks. They will voluntarily sign the informed consent before enrollment. The patients will be free to withdraw from the study at any time without a specific reason and without any penalty or loss of benefits. However, we will attempt to record the reason for withdrawal and encourage the participant to remain in the study if possible.

### Interventions

Both ancient literature and previous clinical studies have provided the validated and reliable acupuncture procedure for treating knee pain. Acupuncture treatment will be performed in different compartments by two study-trained, licensed acupuncturists who have more than 6 years of acupuncture clinical practice experience.

#### Verum acupuncture group

**Fig. 3:**
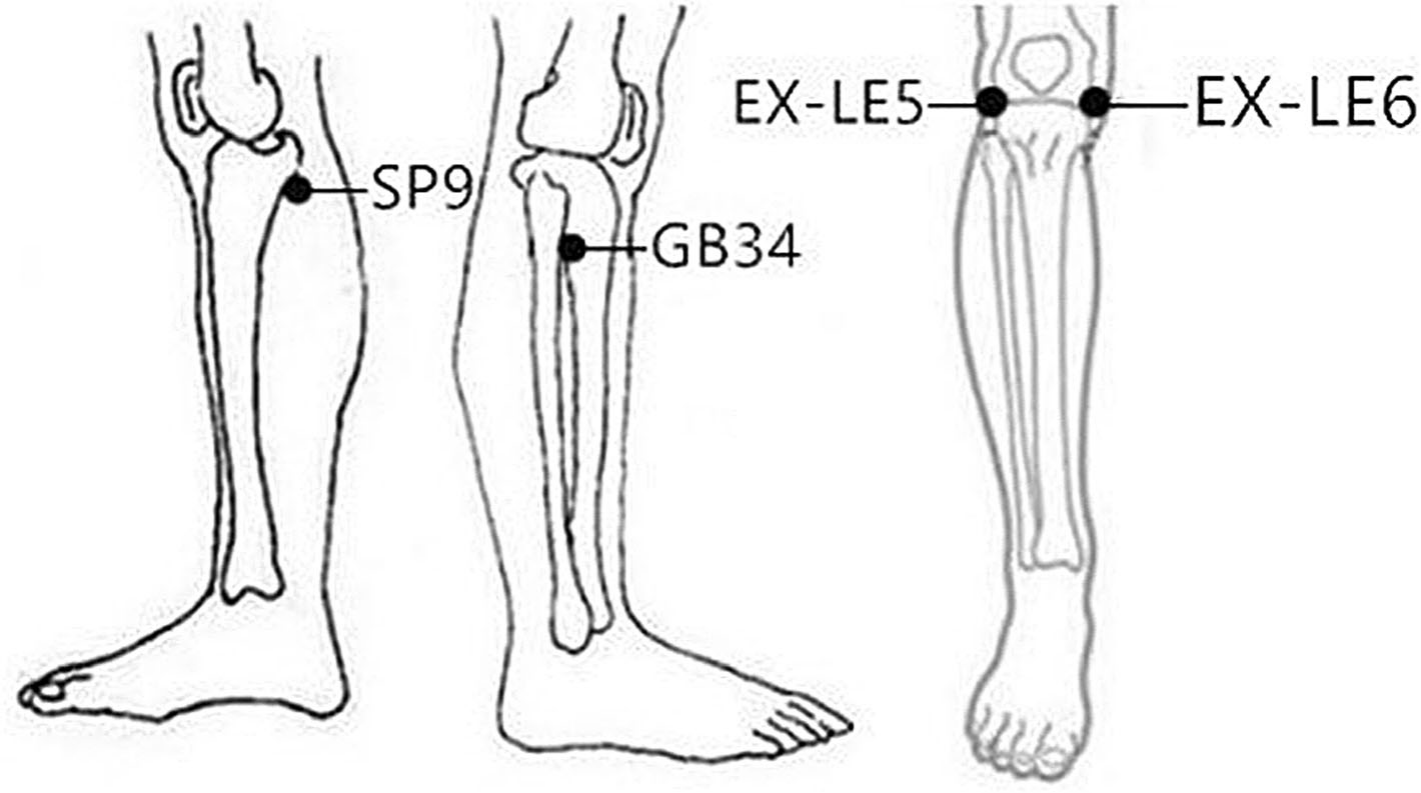
Locations of acupoints: SP9 (*yinlingquan*), on the medial side of the shank, at the depression posterior and inferior to the medial condyle of the tibia. GB34 (*yanglingquan*), on the lateral side of the lower leg, in the depression anterior and inferior to the head of the fibula. EX-LE04 (*neixiyan*), in the depression located on the medial side of the patellar ligament. EX-LE5 (*waixiiyan*), in the depression located on the external side of the patellar ligament

The selected acupoints for the verum acupuncture treatment include GB34 (*yanglingquan*), SP9 (*yinlingquan*) and two *ashi* points. An *ashi* point is the position where there is a sensation of acid, distension and pain around the knee joint. KOA’s *ashi* point is a temporary acupoint which can appear anywhere in the knee joints. If there is no *ashi* point, EX-LE04 (*neixiyan*) and EX-LE5 (*waixiiyan*) will be selected for *ashi*- point replacement (Fig. 3). If the KOA patients have unilateral knee pain, points on the painful knee will be selected. If the KOA patients have bilateral knee pain, points on both knees will be selected.

Referring to previous studies [23, 24], the acupuncture procedures are as follows: after the skin has been cleaned with tincture of iodine and alcohol, sterile acupuncture needles (0.25 mm in diameter, 25 or 40 mm long, Hwatuo, Suzhou, China) will be inserted for 0.5–1.5 *cun* and gently twisted, lifted and thrust with even amplitude, force and speed for four to six times until *deqi* is obtained (soreness, numbness, distension and heaviness). The needles will then be left in place for 30 min, during which manipulation will be applied every 10 min. Every patient will receive five treatments per week for 2 weeks.

#### Sham acupuncture group

**Fig. 4:**
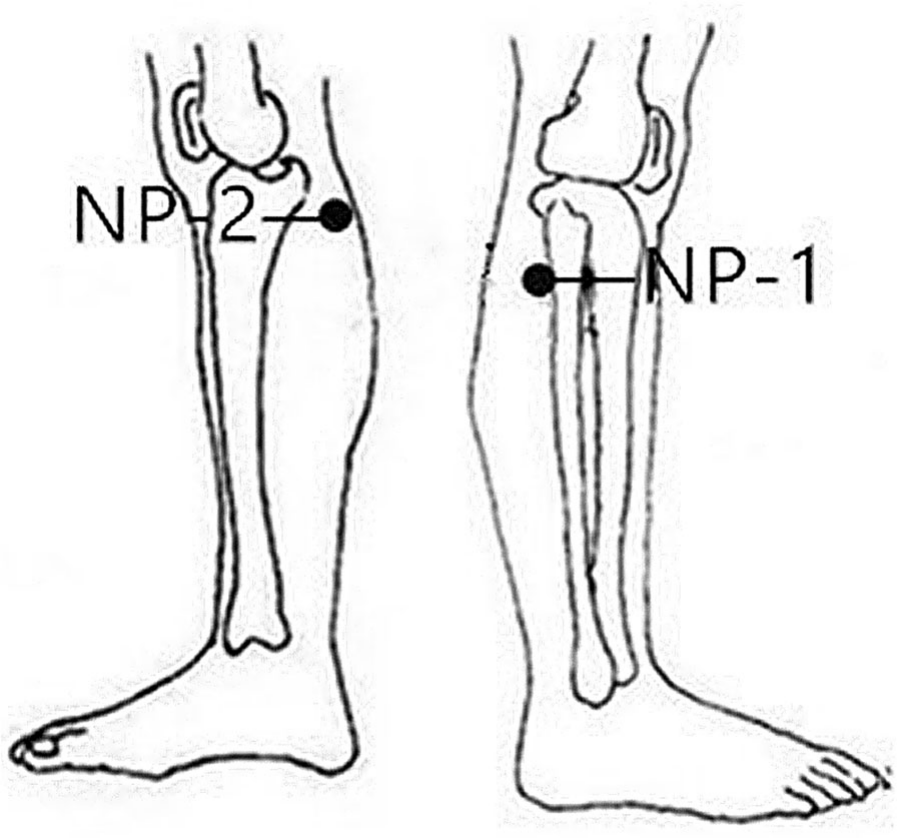
Locations of sham acupoints: NP-1 (sham acupoint 1), 2–3 cm behind GB34. NP-2 (sham acupoint 2), 2–3 cm behind SP9

**Fig. 5:**
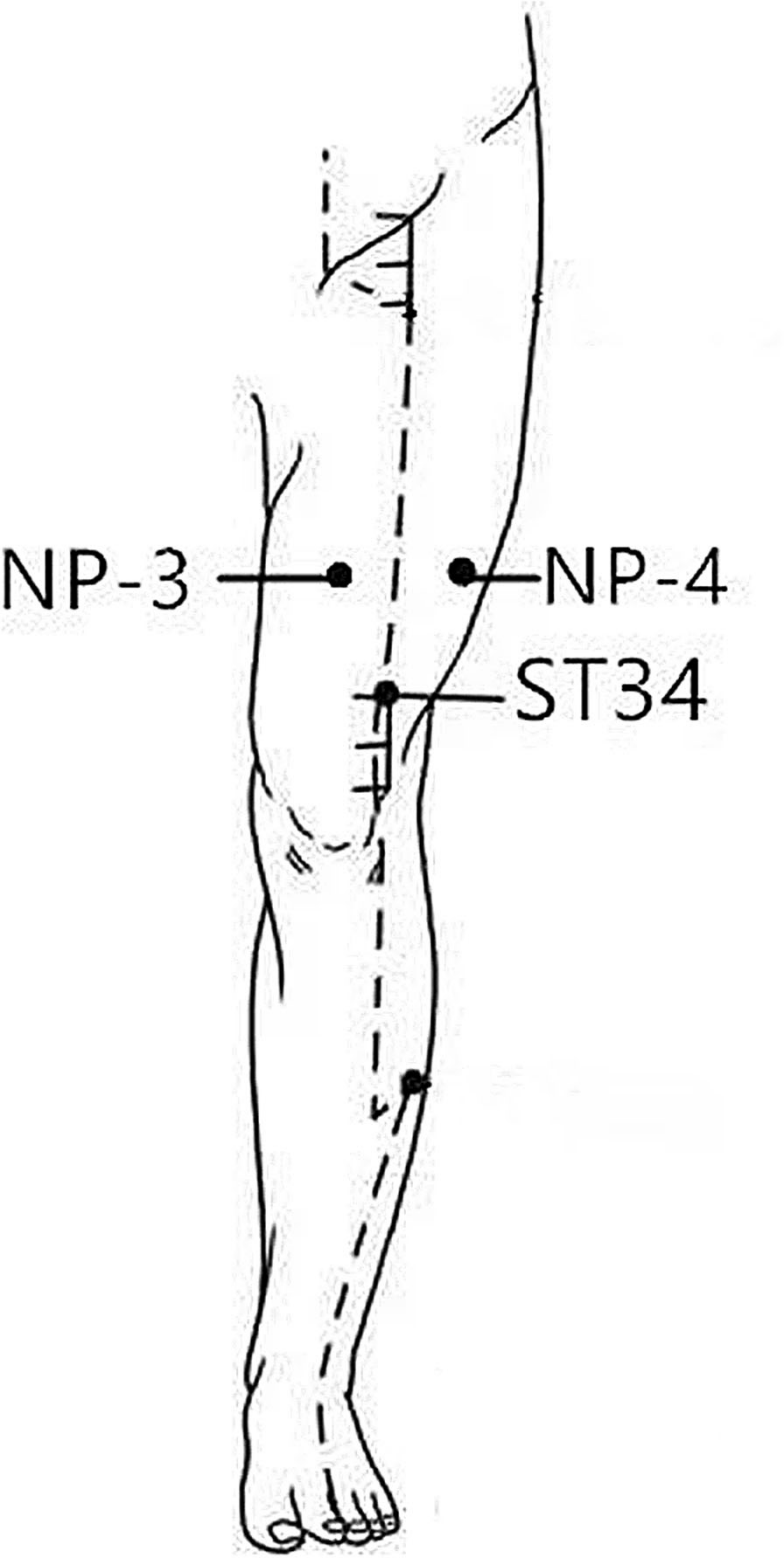
Locations of sham acupoints: NP-3 (sham acupoint 3), 4 *cun* above the base of the patella and the midpoint between the Spleen Meridian and Stomach Meridian. NP-4 (sham acupoint 4), 4 *cun* above the base of the patella and the midpoint between the Gallbladder Meridian and Stomach Meridian

The selected non-acupoints in the sham acupuncture group include NP-1 (2–3 cm behind GB34), NP-2 (2–3 cm behind SP9), NP-3 (4 *cun* above the base of the patella and the midpoint between the Spleen Meridian and Stomach Meridian) and NP-4 (4 *cun* above of base of the patella and the midpoint between the Gallbladder Meridian and Stomach Meridian) (Figs. 4 and 5). The acupuncture procedure is the same as that in the verum acupuncture group.

#### Waiting-list group

In the waiting-list group, participants will receive no treatment within 2 weeks. After 2 weeks’ observation, patients will receive 10 verum acupuncture treatments or 2 weeks’ celecoxib treatment for free as compensation.

#### Concomitant medications and other interventions

Patients will be instructed not to take any caffeine (such as tea and coffee), and not to take any other medications or use interventions such as weight-loss techniques and physical activity, etc. for KOA during this study. In cases of severe knee pain, ibuprofen (300 mg per capsule with sustained release) will be allowed as rescue medication and should be recorded on the knee-pain diary. After the end of the study, patients will be taught weight-loss techniques and physical activity.

Patients are also asked to record the name, dose, date and the exact time of the medications used, and to report to the researcher if they take any regular medications during the study.

#### Patient safety

Adverse events caused by acupuncture, such as pain, bleeding, fainting or other severe events, should be processed immediately and recorded in detail in the case report form.

### MRI data acquisition

MRI data will be acquired with a 3.0-T magnetic resonance scanner (GE 3.0 T MR750, Wauwatosa, WI, USA) with a 16-channel phase-array head coil at the MRI Center at the University of Electronic Science and Technology of China. Participants are asked to stay awake and to keep their heads still during the scan, with their eyes closed and ears plugged. Participants are asked to keep relaxed and not to think of anything particular during the whole scan.

Prior to the blood-oxygen-level-independent (BOLD) resting-state functional images, a high-resolution structural image for each subject will be acquired using a three-dimensional MRI sequence (3DT1) with a voxel size of 1 mm^3^, employing an axial fast spoiled-gradient-recalled sequence (repetition time 6.008 ms, echo time 1.7 ms, data matrix, 256 × 256; field of view, 256 × 256 mm^2^). The BOLD resting-state functional images will be obtained with echo-planar imaging (31 contiguous slices with a slice thickness of 5 mm, repetition time 2000 ms, echo time 30 ms, flip angle 90°, field of view, 240 × 240 mm^2^; data matrix 64 × 64, total volumes 205). After the BOLD-fMRI scan, diffusion tensor imaging (DTI) sequences with single-shot echo-planar imaging will be acquired to detect any possible abnormality of the white matter with the following parameters: field of view 256 × 256 mm^2^, repetition time 8500 ms, echo time minimum, matrix = 128 × 128, slice thickness 2 mm, 78 continuous axial slices with no gap. Two diffusion-weighted sequences will be acquired using gradient values b = 0 s/mm^2^ and b = 1000 s/mm^2^ with the diffusion sensitizing gradients applied along 64 non-linear directions.

### Outcome measurement

The clinical outcome will measure patients’ knee pain and physical function, quality of life (QOL) and emotional status. All measurement will be performed at the baseline and at the end of the treatment. The primary outcome measurements are the Visual Analog Scale (VAS)[25] and short-form of the McGill Pain Questionnaire (SF-MPQ) [26]. The VAS is a 10-point scale selected to quantitatively evaluate the level of knee pain during the study. It scores from “0 (none),” “about 2 (mild),” “around 5 (moderate),” “almost 8 (severe),” and “nearly 10 (unbearable)” spaced along the continuum. The SF-MPQ focuses on evaluating the sensory and affective components of knee pain in the last 2 weeks. It mainly consists of 15 descriptors (11 sensory and four affective) rating on an intensity scale as “0 = none,” “2 = moderate,” and “3 = severe.” A higher total score indicates more severe pain.

The secondary outcomes are the Western Ontario and McMaster Universities Osteoarthritis Index (WOMAC) [27], the Attention Test Scale (ATS), the Pain Assessment of Sphygmomanometer (PAS) [28] and the 12-Item Short Form Health Survey (SF-12) [29]. The WOMAC is a self-administered questionnaire consisting of 24 items divided into three subscales: pain (five items), stiffness (two items) and physical function (17 items). These measurements will be used to subsidiarily evaluate the symptom and QOL improvement at the baseline and the end of treatment.

Furthermore, to investigate the influence of emotional state on the brain activity, the Self-rating Anxiety Scale (SAS) [30] and the Self-rating Depression Scale (SDS) [31] will be used at the baseline and the end of treatment. To ensure the consistency of acupuncture manipulation, the *deqi* scale will be performed after each acupuncture treatment (Fig. 2). The *deqi* scale is derived from the Chinese version of the modified Massachusetts General Hospital Acupuncture Sensation Scale (C-MASS) [32, 33], the validity and reliability of which have been tested [33, 34]. The scale will be used to evaluate the needle sensations (including soreness, numbness, fullness, heaviness, aching), respectively. All the evaluations are evaluated by two trained, licensed physicians.

### Data management and monitoring

The case report form (CRF) includes observation time points, scanning time points, outcome measures, adverse events and safety evaluations. The outcome assessors will be required to follow the requirements of the CRF and fill in the relevant information in a timely and accurate manner. Only outcome assessors have access to the CRFs and perform double-data entry. The Evidence-based Medicine Center of the university will be responsible for monitoring the study as well as the data every 3 months.

### Data analysis

Before the data analyses, the research group will provide a statistical scheme to the statisticians. The scheme will include the required data and processing method. The data analysis process according to the scheme will be completed by statisticians who are independent from the research team and blinded to the test settings.

For the clinical data, statistical analyses will be performed with SPSS 22.0 statistics software (IBM Corp, Armonk, NY, USA). For the fMRI data, preprocessing and functional connectivity analysis will be performed by SPM12 software (SPM12, Wellcome Department of Imaging Neuroscience, London, UK; http://www.fil.ion.ucl.ac.uk/spm/) performing on MATLAB 8.6 (Mathworks, Inc., Natick, MA, USA) .

Both between and within groups, the numerical variables include the clinical data collected from the VAS, the short form of the McGill Pain Questionnaire, the WOMAC, ATS, SAS, SDS, PAS, *deqi* scale and the SF-12 scale, and Fisher’s transformed z value by functional connectivity analysis on neuroimaging data. Since there are three groups (verum acupuncture group, sham acupuncture group and waiting-list group) with two time points (pre-treatment and post-treatment), the repeated measures analysis of variance (ANOVA) will be employed to analyze both the clinical and neuroimaging data generated by the present study design. In the 2 × 3 group factorial design, the dependent variables were the clinical/neuroimaging data collected from pre-treatment and post-treatment, and data in the three different conditions (groups) will serve as the independent variable. Then, *t* tests will be used in the post-hoc analysis. The chi-square test will be used for categorical variables. All data are presented as the mean ± standard deviation (SD). Data are analyzed following intention to treat. A two-sided test is applied for available data and *P* < 0.05 is considered statistically significant. Finally, the Pearson’s correlation between the changes of cerebral activity and the improvement of clinical variables will be performed in each group.

## Discussion

This is a randomized, sham- and waiting-list-controlled fMRI study aiming to explore how longitudinal verum acupuncture treatment works in alleviating knee pain by modulating brain function in KOA patients.

### The reason of acupoint/non-acupoint selection

According to the theory of traditional Chinese acupuncture, all the points can treat the disease of the local area and adjacent area. In this study, four local acupoints are selected in the verum acupuncture group, including GB34 (*yanglingquan*), SP9 (*yinlingquan*) and two *ashi* acupoints around the knee joint. GB34, the *He-sea* point and lower *He-sea* point of the Gallbladder Meridian of Foot-*Shaoyang*, is located on the lateral side of the calf and in the depression below the front of the fibular head. GB34 is also known as the converging point of the tendons and is usually used for knee-pain treatment. SP9 is the *He-sea* point of the Spleen Meridian of Foot-*Taiyin*. It is located on the medial side of the leg, and in the depression between the medial tibial medial margin and the medial tibial margin. It is one of the most commonly used points for the treatment of knee joint diseases. *Ashi* acupoints are the pain spots or the tender points which are good for alleviating local pain. If there is no *ashi* acupoint reported, EX-LE04 and EX-LE05 will be selected as a replacement for the *ashi* acupoints. Briefly, GB34, SP9, *ashi* points, EX-LE04 and EX-LE05, which are also used in many other acupuncture-treating KOA RCTs [35–37], are commonly used for KOA treatment in the acupuncture clinic (Fig. 3). A clinical study has used these acupoints/non-acupoints and shown that the therapeutic effect of acupuncture plus medication is superior to that of simple medication. The results demonstrated that acupuncture is effective in improving KOA patients’ pain severity and other symptoms [38].

The four non-acupoints in the sham acupuncture group are also selected around the knee joint (NP-1, NP-2, NP-3 and NP-4). These non-acupoints are beside the verum acupoints and not on any regular meridians, which could maximally help to blind the patients and exclude the bias of the spinal segmental effect of the acupoints (Figs. 4 and 5).

### The reason for selecting resting-state BOLD-fMRI as the method for detecting the cerebral responses to acupuncture

With the advantages of its high quality of time and spatial resolution, no radiation, rapid imaging velocity, non-invasion and affordable price [39, 40], fMRI has become the most commonly used neuroimaging techniques in acupuncture central mechanism research. It has been reported that fMRI has been used in 779 acupuncture studies before September 2009 [41]. Based on PubMed, 177 acupuncture-fMRI papers in English were published form 1998 to 2017, which represents 81.94% of all the acupuncture-neuroimaging papers published in English.

As the main type of fMRI, BOLD-fMRI is widely applied in acupuncture research. Compared with task-related fMRI, rs-fMRI has the advantage of providing more comprehensive information on the functional architecture of the brain [42]. Using rs-fMRI, some investigators found that acupuncture treatment can influence KOA patients’ brain function and that verum acupuncture has a different cerebral modulation effect compared with sham acupuncture [43]. But their study has some limitations as we mentioned in the introduction part of this article. Thus, in this study, we will have verum acupuncture, sham acupuncture and waiting-list groups, with a much larger sample size in each group (36 patients in each group). The patients in this study will also receive a larger acupuncture treatment dose (10 acupuncture treatments for 2 weeks).

### Quality control is the precondition for the reliability of results

To improve the result reliability of this study, we designed the quality control program from the following aspects: (1) baseline homogeneity: patient selection for the influence of age and handedness on cerebral function and structure have long been investigated, this study restricts the participants to age between 40 to 60 years and to being right-handed; (2) acupuncture manipulation: in this study, all researchers will be trained to well understand the design and process of the trial, the use of the CRF, as well as measurements of quality. To avoid the manipulation difference, two veteran acupuncturists, who received special and standardized training before the trial, carry out all the treatment with the standard operation procedure. In addition, superfluous communication between acupuncturists and patients during acupuncture is forbidden. Besides, trained outcome assessors will be blinded to group allocation and responsible for the outcome assessment; (3) fMRI scan: to ensure the stability of MRI data acquisition, only one technician will perform all scans in the same MRI machine according to the related operation standard and the design of the study in the fixed condition. Furthermore, the unified guidebook will be used to standardize the opinions and behaviors of the researchers. Moreover, during the 24 h before scanning, participants will be asked to maintain their regular lifestyle and avoid overexertion and staying up late. The use of alcohol, tobacco, tea and coffee is prohibited. Before being scanned, the emotional state of each participant will be evaluated via the emotional state assessment scales. During the scan, participants will be asked to close their eyes and use a blindfold and to plug their ears with earplugs, stay awake and not speak; (4) *deqi*: considering the importance of the *deqi* sensation for the acupuncture effect, the needle sensation scale (*deqi* scale) will be measured after each treatment to evaluate the stimuli and *deqi* sensation in the process, which will help to exclude the bias of *deqi*.

### Limitations

It is difficult to blind patients in the waiting group and acupuncturists for no acupuncture intervention will be performed on this group during the study. However, researchers, acupuncturists and outcome assessors are separate and the group allocation will be concealed.

In summary, this fMRI trial is designed to investigate the central mechanism of verum acupuncture in the treatment of KOA by comparing that of sham acupuncture and waiting-list groups, and by analyzing the correlation between the cerebral activity changes and clinical variable changes. We expect that our findings can provide visualization reference for the clinical application of verum acupuncture for KOA management.

### Trial status

The trial is currently in the participant recruitment stage. The trial began recruitment on 18 October 2017. So far, 89 participants have been recruited and 80 participants have completed the intervention. The recruitment is expected to complete on 31 December 2019.

## Abbreviations

3DT1: A three-dimensional MRI sequence with a voxel size of 1 mm
ACC: Anterior cingulate cortex
ACR: American College of Rheumatology
ATS: Attention Test Scale
BOLD: Blood-oxygen-level-independent
CRF: Case report form
DMN: Medial prefrontal-default mode network
DTI: Diffusion tensor imaging
FC: Functional connectivity
fMRI: Functional magnetic resonance imaging
KOA: Knee osteoarthritis
mPFC: Medial prefrontal cortex
MRI: Magnetic resonance imaging
NSAIDs: Non-steroidal anti-inflammatory drugs
PAS: Pain Assessment of Sphygmomanometer
QOL: Quality of life
ReHo: Regional homogeneity
rFPN: Right frontoparietal network
rs-fMRI: Resting-state functional magnetic resonance imaging
SAS: Self-rating Anxiety Scale
SDS: Self-rating Depression Scale
SF-12: 12-Item Short Form Health Survey
VAS: Visual Analog Scale
WOMAC: Western Ontario and McMaster Universities Osteoarthritis Index
